# ﻿*Dryopteris ningqiangensis*, a new species of Dryopteridaceae from Shaanxi, China

**DOI:** 10.3897/phytokeys.269.163893

**Published:** 2026-01-15

**Authors:** Jian-Quan Tang, Yue-Tong Liu, Wei Fu, Hong-Jin Wei, Bin Li, Zheng-Yu Zuo

**Affiliations:** 1 Xi’an Botanical Garden of Shaanxi Province, Botanical Institute of Shaanxi Province, Xi’an, Shaanxi, 710061, China Botanical Institute of Shaanxi Province Xi’an China; 2 Shaanxi Engineering Research Centre for Conservation and Utilization of Botanical Resources, Xi’an, Shaanxi, 710061, China Shaanxi Engineering Research Centre for Conservation and Utilization of Botanical Resources Xi'an China; 3 College of Modern Agriculture and Biotechnology, Ankang University, Ankang, Shaanxi, 725000, China Ankang University Aankang China; 4 Eastern China Conservation Centre for Wild Endangered Plant Resources, Shanghai Chenshan Botanical Garden, Shanghai, 201602, China Shanghai Chenshan Botanical Garden Shanghai China; 5 Center for Interdisciplinary Biodiversity Research & College of Forestry, Shandong Agricultural University, Tai’an, Shandong, 271018, China Shandong Agricultural University Tai’an China

**Keywords:** *Dryopteris varia* complex, nuclear gene, plastome, Shaanxi Province, taxonomy

## Abstract

*Dryopteris ningqiangensis*, a new fern species of Dryopteridaceae from Shaanxi, China, is described and illustrated herein. Morphologically, *D. ningqiangensis* belongs to the *D. varia* complex but is characterized by papyraceous laminae and sparsely spreading, blackish-brown scales on the stipe. Phylogenetic analyses show that *D. ningqiangensis* is of hybrid origin; its maternal parent is probably an undiscovered tetraploid *D. bissetiana*, and its paternal parent is *D. gymnophylla*.

## ﻿Introduction

*Dryopteris* Adanson is the third largest genus in Dryopteridaceae (Zuo et al. 2025) and the fourth largest among all ferns (PPG I 2016). It contains many species of hybrid origin, especially in D. sect. Variae Fraser-Jenkins (Fraser-Jenkins 1986; Lee and Park 2013; Hori et al. 2014; 2018; Wei et al. 2024). In particular, the *D. varia* complex comprises certain species that have originated from distantly related congeners, involving hybridization between D. sect. Variae and D. sect. Acrorumohra (H. Itô) Li Bing Zhang & H. He or D. sect. Erythrovariae H. Itô (Hori et al. 2018; Wei et al. 2024).

During 2020–2024, we conducted surveys of ferns in Shaanxi, China. The surveyed area predominantly covered the Qinling Mountains and the Bashan Mountains. This region, located near South-Central China and recognized as one of the biodiversity hotspots (Myers et al. 2000), harbors a large number of endemic plants. We noticed a population of a taxon growing on limestone crevices within the Hanjiangyuan Scenic Area, Hanzhong City. This taxon coexists with *D. bissetiana* (Baker) C. Chr. and exhibits a high degree of similarity to *D. sacrosancta* Koidz. However, *D. sacrosancta* is endemic to Japan and resulted from hybridization between *D. chinensis* (Baker) Koidz. and *D. hikonensis* (H. Itô) Nakaike (Hori et al. 2014; 2018). Through comprehensive morphological and molecular phylogenetic investigations, including DNA sequencing, we verified that this population of *Dryopteris* represents a novel species, which is herein described as *D. ningqiangensis*.

## ﻿Materials and methods

Field observations were conducted on living plants of the potentially new taxon, and samples were collected for phylogenetic investigations and specimen observations. The specimens used for morphological comparison and the materials employed for phylogenetic analyses followed our recent study (Wei et al. 2024). Two new samples were added (Table 1) to represent the new species.

**Table 1.** Voucher information and GenBank accession numbers of four new samples included in this study.

Taxa	Voucher	Plastome	AK1	gapCP
***D. bissetiana***	*Zuo6780*	PX394601	PX366028; PX366029	PX361095; PX361096
***D. gymnophylla***	*Zuo6787*	PX394602	PX366030	PX361097
***D. ningqiangensis***	*Zuo6775*	PX394603	PX366031; PX366032; PX366033	PX361098; PX361099; PX361100
***D. ningqiangensis***	*Zuo6775-2*	PX394604	PX366034; PX366035; PX366036	PX361101; PX361102; PX361103

Ploidy levels were determined by flow cytometry (BD FACSCalibur, U.S.A.) through measurement of nuclear DNA content (2C value) of young, fresh leaves, with *Zea mays* L. (1C = 2.70 pg) (Bennett and Smith 1976) serving as the reference standard. The reproductive mode was estimated by counting spores in each sporangium. Specifically, 64 spores and 32 spores per sporangium corresponded to sexual and apogamous reproduction, respectively (Walker 1979). At least ten intact sporangia were examined under a mini-microscope (Yuantu 100×, China).

Young leaves of the potentially new taxon were collected from living plants in the field, and total genomic DNA was extracted from 20 mg of silica-gel-dried leaf material using a modified 4× CTAB method (Doyle and Doyle 1987). For plastid genome sequencing, library preparation and Illumina sequencing were performed at the Germplasm Bank of Wild Species, Kunming Institute of Botany, CAS. *De novo* assembly, sequence connection, and annotation were conducted using GetOrganelle v.1.7.0 (Jin et al. 2020), Bandage 0.8.1 (Wick et al. 2015), and Geneious 9.1.4 (Kearse et al. 2012), respectively, with reference to the previously published plastome of *Dryopteris decipiens* (NC_035854). The newly generated plastome sequences were deposited in NCBI (Table 1).

The products of PCR amplification of the low-copy nuclear *AK1* gene (AK4F: 5’-GATGAAGCCATCAAGAAACCA-3’; AKR2: 5’-ATGGATCCAGCGACCAGTAA-3’) (Hori et al. 2021) and *gapCP* (ESGAPCP8F1: 5’-ATYCCAAGYTCAACTGGTGCTGC-3’ and ESGAPCP11R1: 5’-GTATCCCCAYTCRTTGTCRTACC-3’) (Schuettpelz et al. 2008) were cloned and sequenced at Tsingke Biotechnology Co., Ltd., Kunming. A minimum of six colonies were sequenced for each sample. All newly obtained gene sequences were submitted to NCBI (Table 1).

Three datasets were constructed for phylogenetic analyses. The first dataset comprised 42 *rbcL* sequences retrieved from GenBank or extracted from plastomes using Geneious 9.1.4 (Kearse et al. 2012). The other two datasets consisted of 53 nuclear *AK1* sequences and 55 nuclear *gapCP* sequences. Sequence alignment and manual correction were performed using MAFFT v.7.017 (Katoh et al. 2002) and Geneious 9.1.4 (Kearse et al. 2012).

Bayesian inference (BI) and maximum likelihood (ML) analyses were conducted to infer phylogenetic relationships. BI analyses were performed using MrBayes 3.2.6 (Ronquist et al. 2012) with four parallel Markov chain Monte Carlo (MCMC) chains, ten million generations, and sampling every 1000 generations. The first 25% of sampled trees were discarded as burn-in. ML analyses were carried out using IQ-TREE 1.6.12 (Nguyen et al. 2015) under the GTR+R6 model with 5000 ultrafast bootstrap replicates.

## ﻿Results and discussion

**Figure 1.**
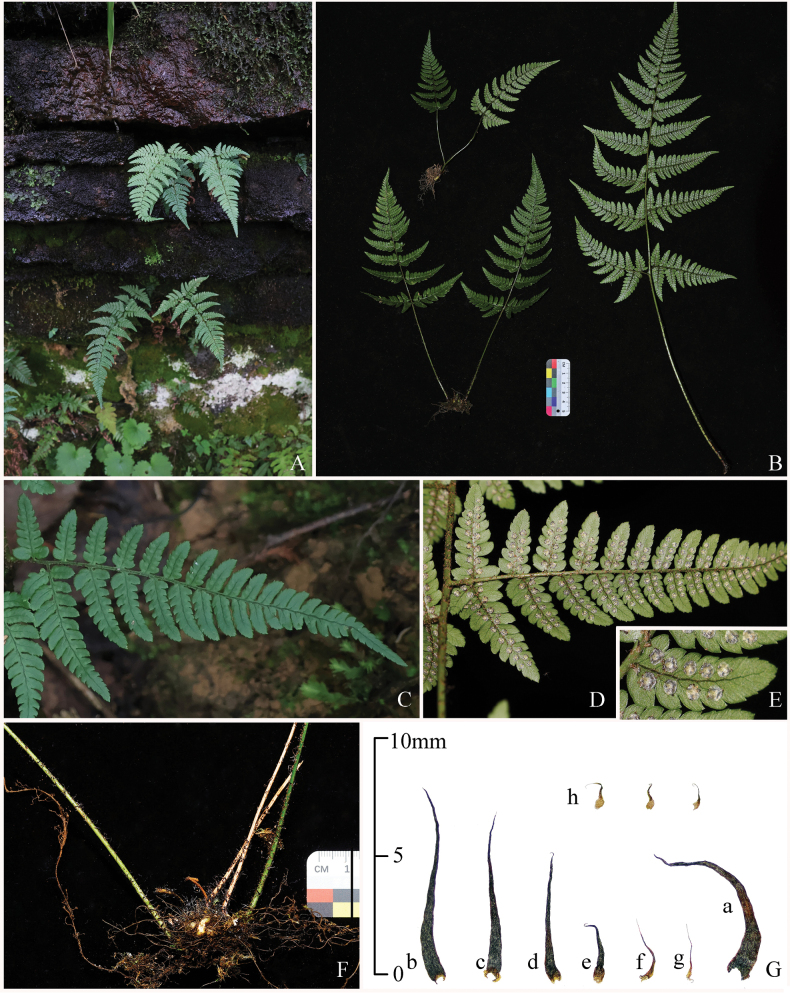
Photos of *Dryopteris ningqiangensis* (Zuo6775). **A.** Habitat; **B.** Habit; **C.** Adaxial view of basal pinna; **D.** Abaxial view of a portion of the lamina; **E.** Sori on ultimate pinnules; **F.** Rhizome with lower portions of stipes; **G.** Scales: **a, b**, scales from rhizome and stipe base; **c–f**, scales from the upper portion of the stipe and rachis; **g, h**, scales from the costa and costule.

**Figure 2.**
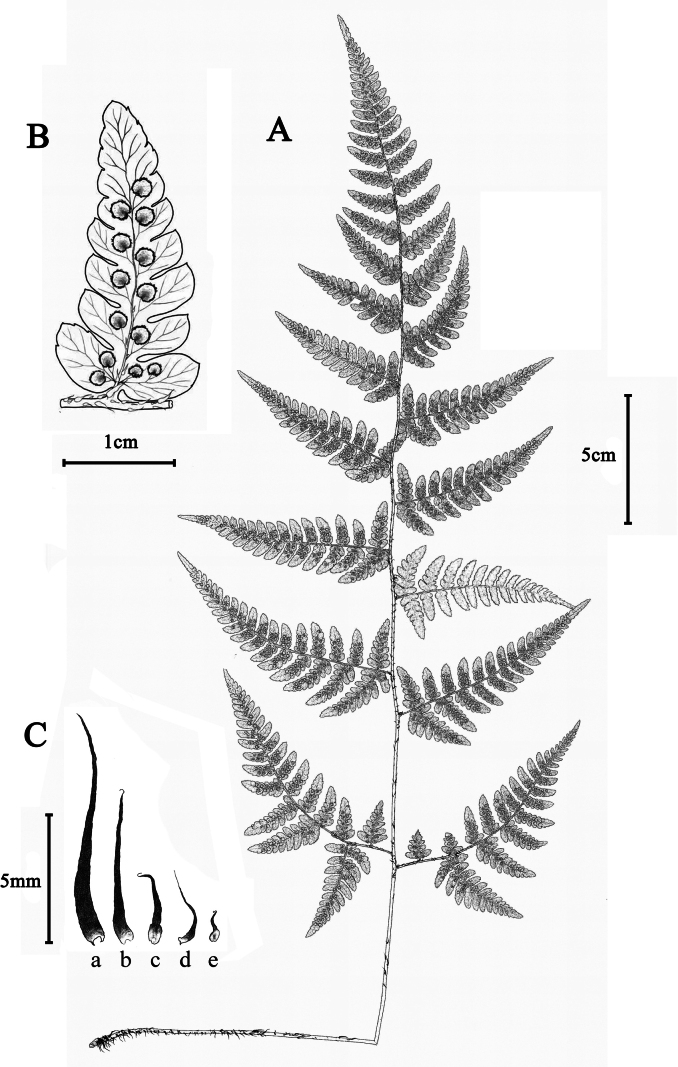
Illustration of *Dryopteris ningqiangensis*. **A.** Habit; **B.** Sori on ultimate pinnules; **C.** Scales: **a**, scales from the rhizome and stipe base; **b–d**, scales from the upper portion of the stipe and rachis; **e**, scales from the costa. (Drawn by Yu-Ting Zhang and Jian Quan-Tang, based on *Zuo6775*).

**Figure 3.**
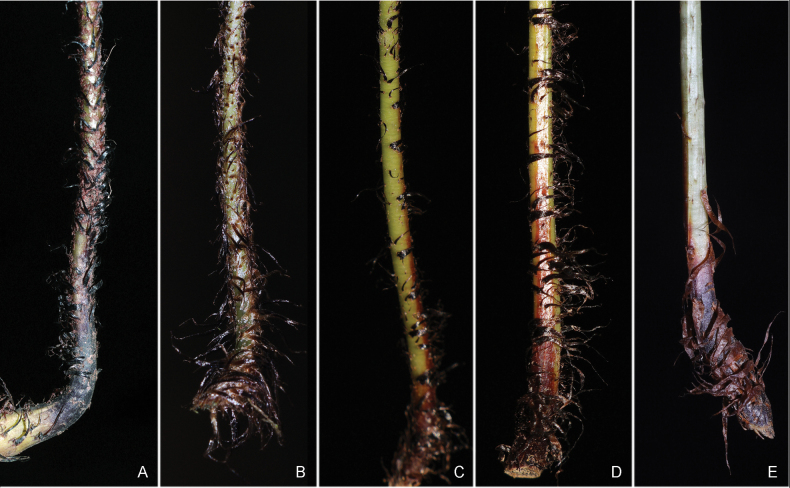
Lower parts of stipes of *D. bissetiana* (**A**, **B**), *D. ningqiangensis* (**C**, **D**), and *D. gymnophylla* (**E**) showing scales.

Morphological comparison showed that *Dryopteris ningqiangensis* has distinct characteristics (e.g., papyraceous laminae, partly deflexed black stipe scales with slightly brown bases, smaller frond size, remotely spaced pinnae, and sori close to the costa) (Figs 1–3) compared with other species, such as *D. bissetiana* and *D. sacrosancta* (Table 2).

**Table 2.** Morphometric comparison of *Dryopteris ningqiangensis* and related species.

Characters	*D. bissetiana*	*D. ningqiangensis*	*D. sacrosancta*	*D. gymnophylla*	*D. protobissetiana*	*D. saxifraga*
**Scales on rachis**	Ascending or deflexed, black, base markedly brown	Spreading, partly deflexed, black, base slightly brown	Spreading, black, base markedly brown	Spreading, dark brown	Spreading, black, base brown	Deflexed, usually black, rarely brown, base bullate brown
**Texture of fronds**	Subleathery or soft leathery	Papyraceous	Herbaceous	Herbaceous	Soft leathery	Soft coriaceous
**Length of stipe**	10–40 cm	5–17 cm	10–40 cm	30–40 cm	10–30 cm	5–20 cm
**Lamina shape**	Narrowly triangular	Narrowly triangular	Ovate-lanceolate	Narrowly pentagonal	Narrowly triangular	Oblong
**Lamina dissection**	Bipinnate, occasionally tripinnate at base	Bipinnate to tripinnate	Bipinnate to tripinnatifid	Tripinnate-pinnatipartite	Bipinnate, occasionally tripinnate at base	Bipinnate, occasionally tripinnate at base
**Lamina size**	20–50 × 10–30 cm	9–32 × 5–16 cm	20–50 × 10–30 cm	25–40 × 15–38 cm	10–40 × 10–20 cm	5–30 × 5–15 cm
**Lamina color**	Dark green or whitish green	Light green	Yellowish green	Light green	Dark green	Whitish green
**Proximity of pinnae**	Approaching	Remote	Approaching	Approaching	Approaching	Approaching
**Pinnule apex**	Obtuse, incised-serrate	Obtuse, serrate	Acute, entire	Obtuse, serrate	Obtuse, serrate	Obtuse, entire, or sinuate
**Sorus position**	Slightly nearer to costa than to margin	Close to costa	Medial	Medial	Slightly nearer to margin than to costa	Medial

The DNA content of the new species was estimated to be 18.1 ± 0.3 pg, which is very close to that of other triploid species in D. sect. Variae (Wei et al. 2024). All spore counts showed that *D. ningqiangensis* has 32 normal spores per sporangium, indicating that this new species is a triploid apogamous species (Table 3). For other related species (*D. bissetiana*, *D. gymnophylla* (Baker) C. Chr., and *D. varia* (L.) Kuntze) from the same locality, the estimated ploidy levels and reproductive modes were consistent with those reported in previous studies (Table 3).

**Table 3.** Estimated ploidy level and reproductive mode of *Dryopteris ningqiangensis* and related species.

Taxa	Voucher	DNA Content (pg)	Estimated ploidy level	Spores per sporangium	Reproductive mode
***D. bissetiana***	TJQ20250911-01	20.51 ± 0.22	3*x*	32	Apogamous
	TJQ20250911-02	20.09 ± 0.19	3*x*		
	TJQ20250911-03	19.67 ± 0.41	3*x*		
	TJQ20250911-04	19.85 ± 0.25	3*x*		
	TJQ20250911-05	19.47 ± 0.29	3*x*		
	TJQ20250911-06	19.67 ± 0.31	3*x*		
	TJQ20250911-07	19.58 ± 0.35	3*x*		
	TJQ20250911-08	19.44 ± 0.29	3*x*		
	TJQ20250911-10	20.41 ± 0.25	3*x*		
	Zuo6780	20.01 ± 0.27	3*x*		
***D. gymnophylla***	TJQ20250911-11	10.17 ± 0.44	2*x*	64	Sexual
	Zuo6787	10.52 ± 0.37	2*x*		
***D. ningqiangensis***	Zuo6775	18.10 ± 0.30	3*x*	32	Apogamous
***D. varia* (*D. immixta*)**	TJQ20250911-09	21.36 ± 0.15	3*x*	NA	NA

**Figure 4.**
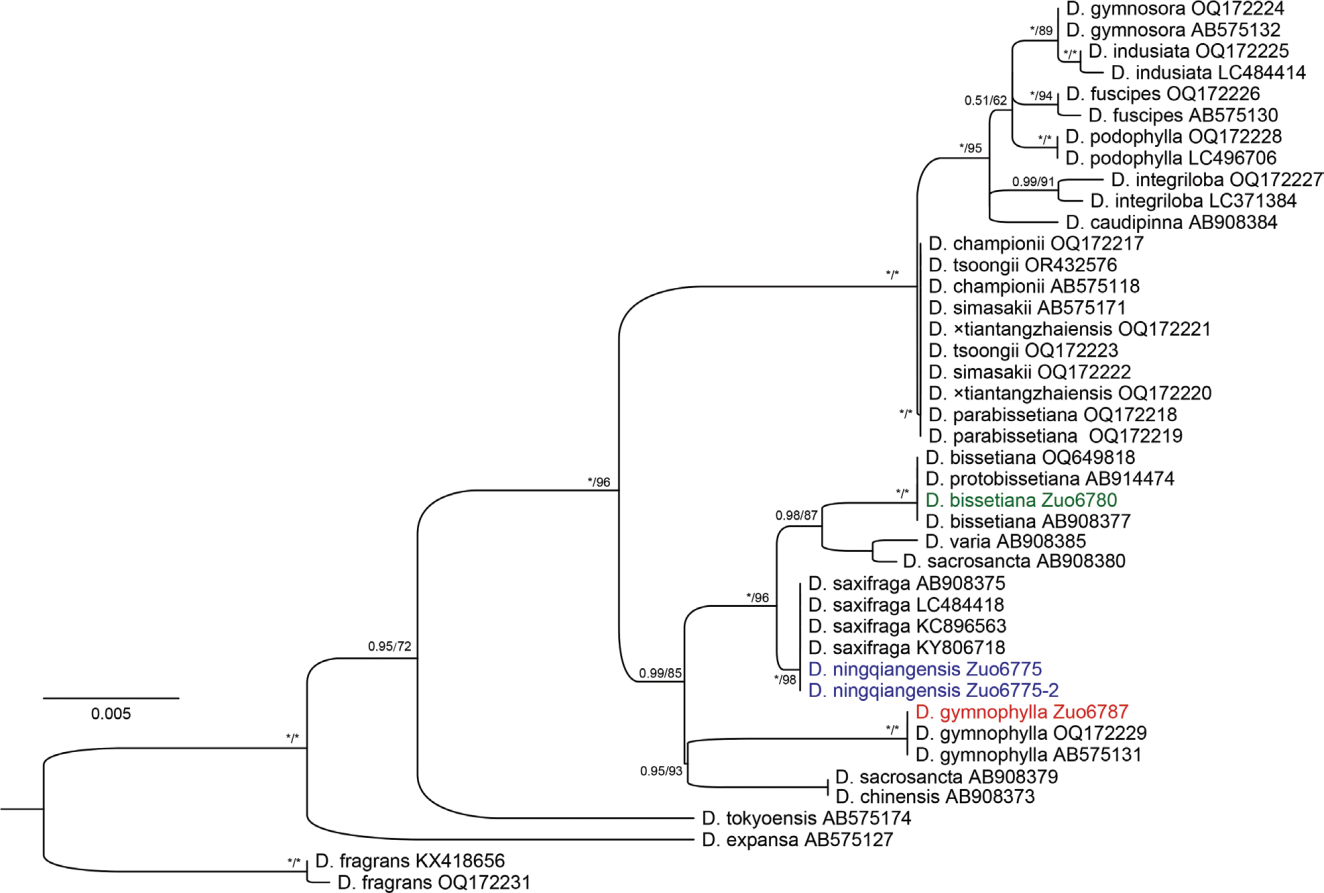
Maximum likelihood phylogram of Dryopteris sect. Variae and related sections based on plastid *rbcL* regions. Support values, including ML ultrafast bootstrap support (UFBS), MP bootstrap support (MPBS), and Bayesian posterior probability (BIPP), are indicated along the branches. An asterisk (*) indicates UFBS/MPBS = 100% or PP = 1.00.

**Figure 5.**
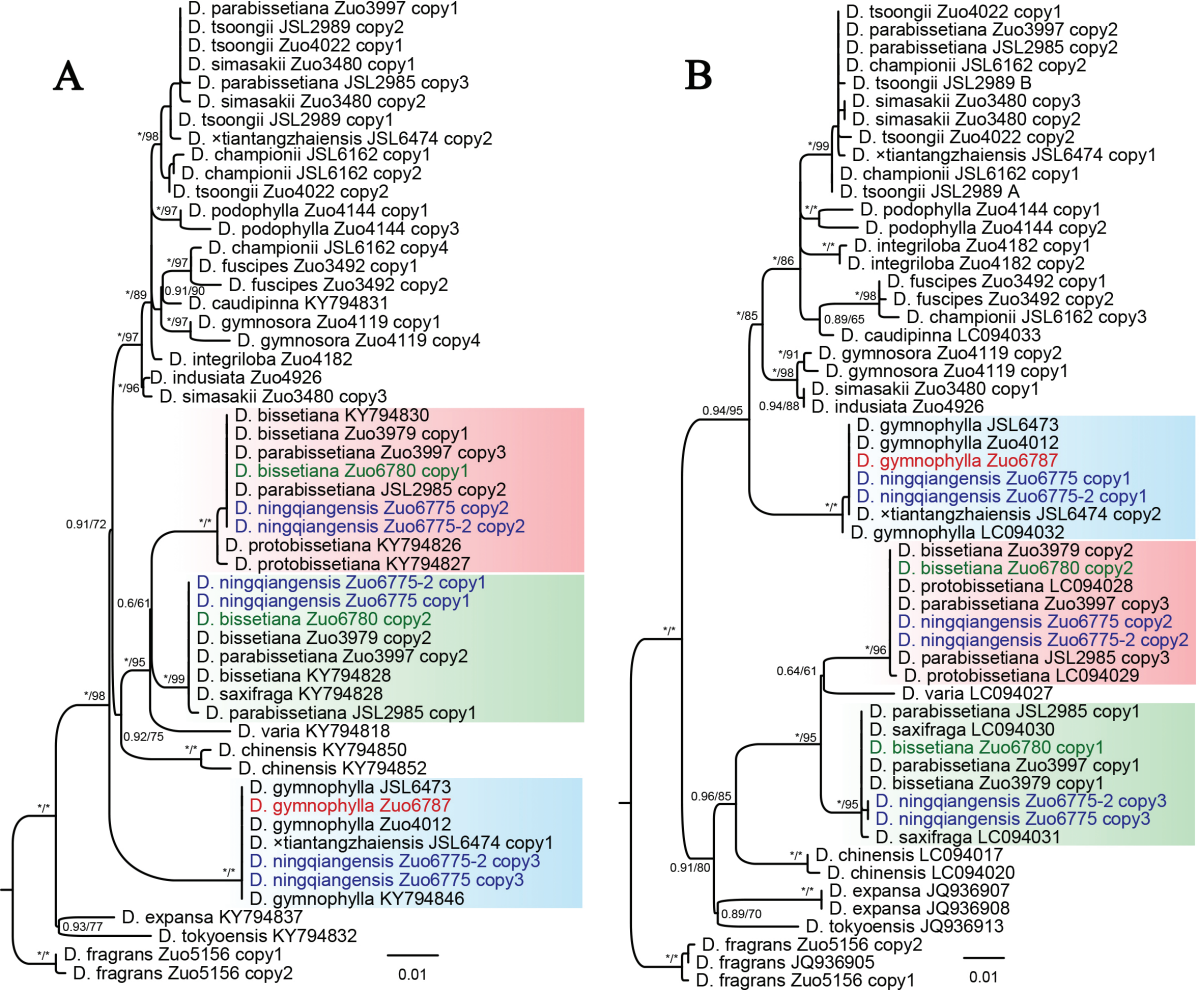
Maximum likelihood phylograms of Dryopteris sect. Variae and related sections based on nuclear genes *AK1* (**A**) and *gapCP* (**B**). Support values, including ML MP bootstrap support (MPBS) and Bayesian posterior probability (BIPP), are indicated along the branches. An asterisk (*) indicates MPBS/BIPP = 100% or PP = 1.00.

Phylogenetic analyses based on plastid genes showed that two samples of *D. ningqiangensis* were nested within the clade of *D. saxifraga* H. Itô (Fig. 4). In addition, Hori et al. (2014, 2018) showed that individuals of *D. bissetiana* possess either the plastid genome of *D. protobissetiana* or that of *D. saxifraga*. In phylogenetic trees based on the *AK1* and *gapCP* genes (Fig. 5), *D. ningqiangensis* exhibited three copies allelic to those of *D. gymnophylla*, *D. protobissetiana*, and *D. saxifraga*. Considering that *D. bissetiana* is a hybridogenous species resulting from *D. protobissetiana* and *D. saxifraga*, and that it grows together with the new species, we hypothesized that *D. ningqiangensis* is of hybrid origin between *D. bissetiana* and *D. gymnophylla*.

The process of hybridization should be carefully interpreted. *Dryopteris gymnophylla*, *D. protobissetiana*, and *D. saxifraga* are diploid sexual species, whereas *D. bissetiana* is usually a triploid apogamous species (Hori et al. 2014; Takamiya 1996). In addition, Lee and Park (2013) reported diploid apogamous *D. bissetiana* related to reticulation of triploid apogamous species of the *D. varia* complex, but the material contained only alleles belonging to the clade of *D. saxifraga*. Weng (1989) reported a tetraploid sexual cytotype of *D. bissetiana* from China, but recent studies have shown that *D. bissetiana* appears to be usually triploid apogamous in China (Wei et al. 2024). In this study, we attempted to find diploid or tetraploid individuals of *D. bissetiana*, but all samples examined were triploid (Table 3). From another perspective, some studies have suggested that genome reduction in triploid apogamous species might produce diploid offspring through irregular meiosis during sporogenesis (Lin et al. 1992; Hori et al. 2014). The similar genome constitution of nuclear *AK1* and *gapCP* genes implies that irregular meiosis does not seem to have occurred, or that diploid offspring of triploid *D. bissetiana* coincidentally retained the whole or main genomes of *D. protobissetiana* and *D. saxifraga*. This problem commonly exists in both the *D. erythrosora* complex and the *D. varia* complex (Hori et al. 2014, 2018). The most widely accepted hypothesis is that tetraploid sexual *D. bissetiana* once occurred in the region but is now extinct or rarely encountered.

All results support the conclusion that *D. ningqiangensis* is of hybrid origin between *D. bissetiana* and *D. gymnophylla*. *Dryopteris bissetiana* has been reported as one of the parents of several hybrid-origin species, e.g., *D. kobayashii* Kitag. (= *D. bissetiana* × *D. chinensis*; Hori et al. 2014) and *D. parabissetiana* H.J. Wei & Z.Y. Zuo (= *D. tsoongii* Ching × *D. bissetiana*; Wei et al. 2024). Field observations confirmed that *D. ningqiangensis* and its putative parents occur in the same mountain range, sometimes growing together with the putative maternal parent, *D. bissetiana*. *Dryopteris ningqiangensis* exhibits traits predominantly inherited from the maternal parent, *D. bissetiana*, with some mixed characteristics from both parents. For example, the number of scales in *D. ningqiangensis* is intermediate between those of *D. bissetiana* and *D. gymnophylla* (Fig. 3). Most species in D. sect. Variae are subleathery or softly leathery, except for those of hybrid ori­gin from distantly related congeners, such as *D. erythrovaria* K. Hori & N. Murak. (= *D. hikonensis* (H. Itô) Nakaike × *D. caudipinna* Nakai; papyraceous), *D. kobayashii* (herbaceous), and *D. sacrosancta* (= *D. hikonensis* × *D. chinensis*; herbaceous) (Hori et al. 2018). In these cases, one parent belongs to D. sect. Acrorumohra or D. sect. Erythrovariae, resulting in papyraceous or herbaceous hybrid species. *Dryopteris ningqiangensis* exhibits a similar hereditary pattern, with a papyraceous frond texture resembling that of its paternal parent, *D. gymnophylla*, providing further evidence of its hybrid origin. These reticulate relationships correspond well to the concept of D. sect. Polystichodrys H. Itô (Itô 1939). Itô (1939) suggested that D. sect. Polystichodrys includes D. subsect. Erythrovariae H. Itô, D. subsect. Formosanae H. Itô, and D. subsect. Gymnosorae H. Itô. Itô (1939) additionally placed *D. chinensis* and *D. gymnophylla* in D. subsect. Erythrovariae, together with major members of the *D. erythrosora* complex and the *D. varia* complex distributed in Sino-Japanese regions. Compared with the plastid phylogeny of Zhang et al. (2012), Itô’s (1939) circumscription of D. sect. Polystichodrys is not monophyletic but effectively recognizes the complex reticulate relationships among taxa of the two complexes, with the distantly related *D. chinensis* and *D. gymnophylla* forming a distinct lineage within the genus *Dryopteris*.

## ﻿Taxonomic treatment

### ﻿ Dryopteris ningqiangensis

Taxon classificationPlantaePolypodialesDryopteridaceae

Z.Y. Zuo & J.Q. Tang sp. nov.

E47E2B1D-B639-5C55-96A7-1D99EA736D7F

urn:lsid:ipni.org:names:77375066-1

Figs 1, 2 In Chinese 宁强鳞毛蕨 (níng qiáng lín máo jué).

#### Type.

China • Shaanxi: Ningqiang County, the Hanjiangyuan Scenic Area, 32°45'N, 106°13'E, elev. 1280 m, 30 Aug. 2024, *Zuo6775* (holotype, KUN!; isotypes: KUN!, CSH!, SDFS!, SDFS!).

#### Diagnosis.

*Dryopteris ningqiangensis* is similar to *D. bissetiana* and *D. sacrosancta* in overall shape but differs by the combination of the following characteristics: smaller frond size, partly deflexed black stipe scales on with slightly brown base, papyraceous lamina, remotely spaced pinnae, sori close to costa.

#### Description.

***Plants*** terrestrial, evergreen, 13–46 cm tall. ***Frond*** caespitose. ***Rhizome*** erect or slightly ascending, clothed with dense scales. ***Scales*** linear-lanceolate, flat, spreading, base slightly brown, upper black. ***Stipe*** stramineous, ca. 4–17 cm, sparsely covered with scales, partly deflexed. ***Lamina*** papery, narrowly deltoid, widest at base, acuminate at apex, ca. 9–32 × 5–16 cm, binpinnate to tripinnate, with smaller scales on abaxial surface. ***Pinnae*** remotely spaced, 9–16 pairs, stalked, stalks ca. 0.3–1 cm, apex acuminate, basal 1–2 pairs opposite, upward alternate, basal pair largest, deltoid-lanceolate, up to 9 × 4 cm. ***Pinnules*** 5–14 pairs, margin serrate to pinnate, lanceolate to oblong, base symmetrical, apex obtuse, serrate; basal basiscopic pinnule of basal pinna largest, ca. 1–4 × 0.4–1.7 cm, widest at base, 2-pinnate. ***Rachis and costa*** clothed with linear lanceolate scales and bullate scales. ***Veins*** pinnate, veinlets simple or forked, visible abaxially. **Sori** close to costule, in 1 row on either side of costule. ***Indusia*** brown, reniform, margins entire. Reproductive mode and ploidy level: triploid apogamous.

#### Ecology and geographical distribution.

*Dryopteris ningqiangensis* was presently only known from Ningqiang County, Shaanxi Province, China. It was found growing mostly on shaded cliffs and sometimes on the foot of rocks in mountain forests at an elevation of 1100–1400 m.

#### Etymology.

The epithet is taken from Chinese Pinyin “ningqiang”, the name of the county in western Shaanxi Province, located at the junction of the Qinling and Bashan Mountains.

## Supplementary Material

XML Treatment for Dryopteris ningqiangensis

## ﻿Additional information

### Use of AI

No use of AI was reported.

### Author ORCIDs

Jian-Quan Tang https://orcid.org/0009-0002-8403-0989

Hong-Jin Wei https://orcid.org/0000-0002-3679-6683

Bin Li https://orcid.org/0000-0001-9254-8003

Zheng-Yu Zuo https://orcid.org/0000-0002-8334-6132

### Data availability

All of the data that support the findings of this study are available in the main text.
